# Impact of the *APOBEC3A/B* deletion polymorphism on risk of ovarian cancer

**DOI:** 10.1038/s41598-021-02820-z

**Published:** 2021-12-06

**Authors:** Liv B. Gansmo, Nigar Sofiyeva, Merete Bjørnslett, Pål Romundstad, Kristian Hveem, Lars Vatten, Anne Dørum, Per E. Lønning, Stian Knappskog

**Affiliations:** 1 K.G. Jebsen Center for Genome-Directed Cancer Therapy, Department of Clinical Science, University of Bergen, 5021 Bergen, Norway; 2 Department of Oncology, Haukeland University Hospital, Bergen, Norway; 3 Department of Molecular Oncology, Oslo University Hospital Radium Hospitalet, Oslo, Norway; 4 Institute for Cancer Research, University of Oslo, Oslo, Norway; 5 Department of Public Health, Faculty of Medicine, Norwegian University of Science and Technology, Trondheim, Norway; 6 K.G. Jebsen Center for Genetic Epidemiology, Department of Public Health, Faculty of Medicine, Norwegian University of Science and Technology, Trondheim, Norway; 7 Department of Gynecologic Oncology, Oslo University Hospital, Norwegian Radium Hospital, Oslo, Norway

**Keywords:** Cancer, Ovarian cancer

## Abstract

A germline 29.5-kb deletion variant removes the 3’ end of the *APOBEC3A* gene and a large part of *APOBEC3B*, creating a hybrid gene that has been linked to increased APOBEC3 activity and DNA damage in human cancers. We genotyped the *APOBEC3A/B* deletion in hospital-based samples of 1398 Norwegian epithelial ovarian cancer patients without detected *BRCA1/2* germline mutations and compared to 1,918 healthy female controls, to assess the potential cancer risk associated with the deletion. We observed an association between *APOBEC3A/B* status and reduced risk for ovarian cancer (OR = 0.75; CI = 0.61–0.91; *p* = 0.003) applying the dominant model. Similar results were found in other models. The association was observed both in non-serous and serous cases (dominant model: OR = 0.69; CI = 0.50–0.95; *p* = 0.018 and OR = 0.77; CI = 0.62–0.96; *p* = 0.019, respectively) as well as within high-grade serous cases (dominant model: OR = 0.79; CI = 0.59–1.05). For validation purposes, we mined an available large multinational GWAS-based data set of > 18,000 cases and > 26,000 controls for SNP rs12628403, known to be in linkage disequilibrium with the *APOBEC3A/B* deletion. We found a non-significant trend for SNP rs12628403 being linked to reduced risk of ovarian cancer in general and similar trends for all subtypes. For clear cell cancers, the risk reduction reached significance (OR = 0.85; CI = 0.69–1.00).

## Introduction

Epithelial ovarian cancer (OC) is the third most common gynaecologic cancer and accounts for an estimated 300,000 new cases and roughly 185,000 deaths each year worldwide^1^. As such, ovarian cancer is the gynaecological cancer with the worst prognosis and highest mortality rate^2^. OC is not a single disease but consists of subtypes that can be classified based on distinctive morphologic and molecular genetic features^3^. Although high penetrance germline mutations in homology-directed repair genes such as *BRCA1* and *BRCA2*^4,5^ are well described, these mutations only account for about 10% of the cases^6^. The remaining fraction of genetic risk factors is assumed to be low-penetrance risk alleles.

Over the last decade, advantages in deep sequencing technologies have revealed the complexity of cancer evolution; the mutational landscape of multiple human cancer types has been described and mutational signatures have been identified, casting light on mutational processes driving tumour evolution and adaption^7–9^. As a result of this, a growing number of studies have started to link the apolipoprotein B mRNA editing enzyme catalytic-polypeptide-like (APOBEC) family of cytidine deaminases to specific innate enzymatic mutational processes in human cancers^7,10–13^.

The APOBEC3 subfamily of proteins (APOBEC3A-G) is known for their ability to protect human cells from viral infections by introducing mutations in single-stranded nucleic acids^14^. However, APOBEC3B has also been reported to edit genomic DNA^15^, while APOBEC3A can hypermutate nuclear DNA, creating double-stranded DNA breaks^16,17^. In addition, elevated *APOBEC3B* expression has been found to correlate with total mutation load in a limited number of ovarian cancer patients^18^ and to predict both worse overall- and disease-free survival^19^.

A common germline deletion of 29.5-kb in the *APOBEC3* genes removes the 3′ end of the *APOBEC3A* gene and a large part of *APOBEC3B*. This deletion creates a hybrid gene transcribing an mRNA containing the *APOBEC3A* coding region and the *APOBEC3B* 3′ UTR^20^. The hybrid mRNA has been found to be more stable than the wild-type and may thus lead to increased intracellular levels and subsequent higher DNA damage caused by APOBEC3 activity^21^. In line with this, the *APOBEC3A/B* deletion variant has been linked to hypermutator phenotypes and the presence of ABOPEC-related mutational signatures in breast cancer^11,22^. Interestingly, the *APOBEC3A/B* deletion variant is more common among individuals of Asian ancestry compared to European ancestry, with a minor allele frequency of 37% and 6% respectively^20^.

Given the link to specific mutational processes, several studies have assessed whether the *APOBEC3A/B* deletion variant may confer cancer risk. So far, case–control studies have shown that the *APOBEC3A/B* deletion is associated with a moderately increased risk for breast cancer in women of Asian descent^23–25^, while the findings among women of European descent are conflicting: Xuan et.al reported the *APOBEC3A/B* deletion variant to be associated with increased risk for breast cancer in Europeans^26^, however, subsequent studies did not reproduce these findings^27–29^. While Qi and co-workers reported the *APOBEC3A/B* deletion variant to be associated with OC among Chinese women^30^, a lack of such association was observed among OC patients in the European population^29^.

In the present study, we performed a case–control study in a large Norwegian hospital-based sample set and previously analysed population-based controls, in order to assess the potential association between the *APOBEC3A/B* deletion variant and risk of ovarian cancer.

## Methods

### Study population

All cases included in this case–control study were obtained from hospital-based cohorts of Norwegian patients diagnosed with OC at Oslo University Hospital, Radiumhospitalet (n = 1611). These cases have been used for genotype analyses of other polymorphisms previously^31,32^. Among these cases, 213 were found to harbour *BRCA1* (n = 147) or *BRCA2* (n = 66) germline mutations. Only those patients without detected *BRCA1/2* mutations were included for the present analyses. Thus, the sample set of OC cases genotyped for *APOBEC3A/B* deletion in the present study consisted of 1398 patients. For comparison (controls), we used data from a previously published analysis of the *APOBEC3A/B* deletion in the Norwegian population^27^. In brief, blood samples from healthy female controls (n = 1918) were drawn from the population-based Cohort of NORWAY^33^, according to selection criteria described previously^27,34^.

All experiments presented in this study were performed according to the Norwegian guidelines for research on human samples and written informed consent to use the samples for research purposes was obtained from all sample donors. The study was approved by the Regional Committees for Ethics in Medical Research of the Central- and South-Eastern Norwegian Health Regions. All methods were performed in accordance with the guidelines for medical research in the above mentioned Heath Regions, the University of Bergen and Haukeland University Hospital, Norway.

### Samples size and statistical power

Given the limited available information for *APOBEC3A/B* genotype, we based power estimates on the positive study by Qi et al*.*^30^. Applying their observed frequencies and an alpha-value of 0.05, a 1-beta value of 0.9 would require a sample size of equal groups of cases and controls of n = 860. Increasing alpha to 0.01 would require equal groups of n = 1210. As such, we found our sample of 1398 cases and 1918 controls to be adequate.

### *APOBEC3A/B* ins/del genotyping

The germline *APOBEC3A/B* deletion was genotyped using quantitative PCR high-resolution melting (qPCR-HRM) curves for the wild-type allele and the deletion allele on a LightCycler 480 II instrument (Roche Diagnostics, Basel, Switzerland) where the melting curve analyses were performed on the Melt Curve Genotyping module in the LightCycler 480 II software version 1.5 (lifescience.roche.com/en_no/products/lightcycler14301-480-software-version-15.html), as previously described^27^. The wild type and deletion allele were genotyped in separate assays using specific primers and hybridization probes for each genotype (Supplementary Table S1). For both assays the qPCR was performed in a total volume of 10 µl, containing 3 mM MgCl_2_, 1 µl LightCycler FastStart DNA Master HybProbe mix (Roche Diagnostics, Basel, Switzerland), 0.125 µM of each probe and either 0.5 µM or 0.1 µM of each primer pairs, for the wild-type allele and the deletion allele, respectively. In the wild-type assay, 0.05 U of Taq DNA polymerase (VWR) was added. The thermocycling settings were 10 min initial denaturation, followed by 45 or 50 cycles of denaturation at 95 °C for 15 s, annealing at 55 °C or 59 °C and elongation at 72 °C for 15 s or 25 s for the deletion and wild-type allele, respectively. The high-resolution melting had an initial denaturation at 95 °C for 30 s, followed by melting from 40 °C to 80 °C with a ramp rate of 0.19 °C/sec ending with a cooling step at 40 °C for 30 s.

For validation purposes and to call genotypes in samples with ambiguous results in the qPCR-HRM-assay, 300 out of the 1398 ovarian cancer samples (21%) were also genotyped for a SNP (rs12628403) in close proximity to *APOBEC3A* and *B*, and in strong linkage disequilibrium with the deletion allele. This SNP was genotyped using a custom-made LightSNiP assay (TIB Molbiol GmbH, Berlin, Germany) according to the manufactures instructions as described previously^27^. In brief, in a final reaction volume of 10 µl containing 3 mM MgCl_2_, 1 µl of LightCycler FastStart DNA Master HybProbe mix (Roche Diagnostics), 0.5 µl LightSNiP mix (TIB MOLBIOL) were mixed with 10–50 ng DNA. The thermocycling was set as follows; 10 min initial denaturation at 95 °C, followed by 45 cycles of denaturation at 95 °C for 10 s, then an annealing and elongation step for 10 s at 60 °C and for 15 s at 72 °C, respectively. The subsequent melt curve conditions were started with an initial denaturation at 95 °C for 30 s, followed by melting from 40 °C to 75 °C with a ramp rate of 0.19 °C/sec and a final cooling step for 30 s at 40 °C. Zero out of the 300 samples analysed for SNP rs12628403 were found to have another *APOBEC3A/B* deletion genotype than expected, based on the known linkage between the two loci. Thus, our present data indicated a recombination rate of 0%, which is in line with previous observations of recombination rates in European populations^27,35^. Thus, the risk of recombination causing erroneous genotyping in those cases where SNP rs12628403 guided interpretation of ambiguous qPCR-HRM results, was considered negligible.

### Data mining (GWAS)

For validation purposes, we mined available data from the results summary of the Ovarian Cancer Association Consortium (OCAC)^36^ at ocac.ccge.medschl.cam.ac.uk/data-projects/. Given that the *APOBEC3A/B* deletion was not called in this data set, we mined the data for the summary results of SNP rs12628403 instead and used this as a mark for the *APOBEC3A/B* deletion allele (see explanation about linkage disequilibrium, above). Genotype information for individual samples was not available.

### Statistical analysis

Potential associations between the *APOBEC3A/B* deletion variant and risk for ovarian cancer were assessed by estimating odds ratios (ORs) with 95% confidence intervals (CIs) and Chi-square tests. Comparisons of continuous variables between groups were performed by Mann–Whitney rank tests. All *p*-values are given as two-sided. All statistical analyses were performed using the STATA software v.16.1 (StataCorp. 2019. Stata Statistical Software: Release 16. College Station, TX: StataCorp LLC.; www.stata.com).

## Results

### Distribution of *APOBEC3A/B* genotypes

The genotype distribution of the *APOBEC3A/B* deletion variant in healthy female controls and OC patients is listed in Table 1. For the sample of 1918 healthy female controls, we have previously reported the genotype distribution of the deletion to be in Hardy–Weinberg equilibrium (*p* values > 0.4), and a minor allele frequency (MAF) of 0.094^27^. Similarly, in the present analysis, we found the distribution among our 1,398 OC cases to be in Hardy–Weinberg equilibrium (*p* = 0.386), with a MAF of 0.072.

**Table 1:** *APOBEC3A/B* genotype distribution and risk estimates for ovarian cancer.

Cases/controls				Dominant model	Recessive model	Allele model
	Genotype *APOBEC3A/B* n (%)			OR (95% CI)	OR (95% CI)	OR (95% CI)
	ii^1^	id^2^	dd^3^	dd + id vs ii	dd vs id + ii	d vs i
Healthy control^4^	1576 (82.2)	323 (16.8)	19 (0.99)	-	-	-
Ovarian cancers	1203 (86.1)	190 (13.6)	5 (0.36)	0.75 (0.61–0.91) *p* = 0.003	0.36 (0.10–0.99) *p* = 0.034	0.74 (0.62–0.89) *p* = 0.001
Non-serous	372 (86.9)	55 (12.9)	1(0.2)	0.69 (0.50–0.95) *p* = 0.018	0.23 (0.31–1.48) *p* = 0.123	0.69 (0.50–0.92) *p* = 0.011
Clear cell	56 (83.6)	11 (16.4)	0 (0)	0.91 (0.42–1.77) *p* = 0.766	NA -	0.86 (0.41–1.61) *p* = 0.639
Endometrioid	122 (89.1)	15 (10.9)	0 (0)	0.57 (0.30–0.99) *p* = 0.040	NA -	0.56 (0.30–0.95) *p* = 0.029
Mucinous	57 (86.4)	8 (12.1)	1 (1.5)	0.73 (0.31–1.50) *p* = 0.380	1.54 (0.04–9.97) *p* = 0.675	0.79 (0.37–1.52) *p* = 0.476
Serous	828 (85.6)	135 (14.0)	4 (0.4)	0.77 (0.62–0.96) *p* = 0.019	0.42 (0.10–1.25) *p* = 0.100	0.77 (0.62–0.94) *p* = 0.010
High-grade serous	425 (85.3)	71 (14.3)	2 (0.4)	0.79 (0.59–1.05) *p* = 0.095	0.40 (0.05–1.68) *p* = 0.207	0.78 (0.60–1.02) *p* = 0.065

^1^Genotype ins-ins.

^2^Genotype ins-del.

^3^Genotype del-del.

^4^Previously published data (27).

### *APOBEC3A/B* genotypes and risk for ovarian cancer

**Figure 1:**
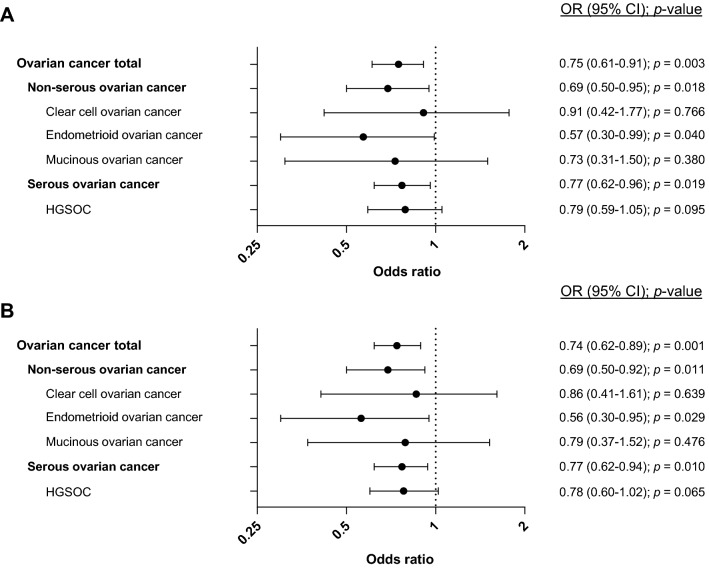
*APOBEC3A/B* deletion and ovarian cancer risk. Forest plots illustrating odds ratios (ORs) with 95% confidence intervals (CI) for ovarian cancer and subtypes, related to the *APOBEC3A/B* deletion variant, applying A) the dominant model and B) the allele model.

Assessing the impact of the *APOBEC3A/B* deletion variant on OC risk, we calculated odds ratios (ORs) as compared to the healthy female controls. Applying the dominant model for the minor allele (*APOBEC3A/B* genotypes del/del + ins/del versus ins/ins), we observed a significant association between *APOBEC3A/B* status and a reduced risk for OC (OR = 0.75; CI = 0.61–0.91; *p* = 0.003; Table 1, Fig. 1a). Similarly, a significant association was observed applying the recessive model (*APOBEC3A/B* del/del vs ins/del + ins/ins; OR = 0.36; CI = 0.10–0.99; *p* = 0.034; Table 1, Supplementary Fig. S1) and the allele model (*APOBEC3A/B* del vs ins; OR = 0.74; CI = 0.62–0.89; *p* = 0.001; Table 1, Fig. 1b). Notably, the number of cases with homozygous del/del genotype was limited (n = 5), causing a wide CI in the recessive model.

### *APOBEC3A/B* genotypes and risk in age groups

Given our previous findings for lung cancer, where the risk associated with the *APOBEC3/B* deletion was significantly linked to age^27^, we stratified OC cases and controls into age groups of 10 years interval, in addition to the groups of patients < 50 years and those > 80 years. We found a significant risk reduction among the age groups 50–59 years and 60–69 years applying the dominant model (OR = 0.63; CI = 0.43 – 0.93; *p* = 0.016 and OR = 0.51; CI = 0.34–0.75; *p* = 4 × 10^–4^ respectively; Supplementary Table S2, Supplementary Fig. S2). No association within the other age groups was observed and we found no trend for age effect on the risk estimates (Supplementary Table S2).

### Risk assessment in subtypes of ovarian cancer

We further stratified the OCs according to the main histological subtypes. Among serous ovarian cancers (n = 965), applying the dominant model, we found an OR similar to that in the the overall assessments (OR = 0.77; CI = 0.62–0.96; *p* = 0.019; Table 1, Fig. 1a). This association was not significant when applying the recessive model, but in this model, the number of observations in the smallest group was limited (n = 4). However, the allele model was in line with the dominant model (OR = 0.77; CI = 0.62–0.94; *p* = 0.01; Table 1, Fig. 1b). Restricting the serous OCs to those of high grade (HGSOC (n = 498)), we found similar ORs to those in the total serous group, both in the dominant, recessive, and allele models (0.79, 0.40, and 0.78 respectively), but these associations did not reach statistical significance. For non-serous ovarian cancers (n = 428), we also observed a reduced OR using the dominant model and the allele models (OR = 0.69; CI = 0.50–0.95; *p* = 0.018 and OR = 0.69; CI = 0.50–0.92; *p* = 0.011, respectively; Table 1, Fig. 1) while significance was not reached when applying the recessive model (OR = 0.23; CI = 0.31–1.48; *p* = 0.123). Among the non-serous subtypes, a trend towards reduced risk was seen in all subtypes (clear cell-, endometroid- and mucinous cancers), while significant only for the endometroid subtype (Table 1, Fig. 1).

### Validation in a mined data set

**Figure 2:**
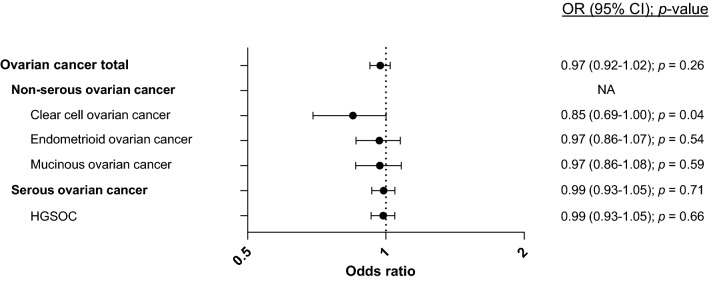
Impact of SNP rs12628403 on ovarian cancer risk in mined data set (OCAC). Forrest plots illustrating odds ratios (ORs) with 95% confidence intervals (CI) for ovarian cancer and subtypes, related to SNP rs12628403. Available data (allele model) mined from the Ovarian Cancer Association Consortium (OCAC) online data set.

We sought to validate our findings in a larger sample set. No such sample set for the Norwegian population was available and we therefore mined the available multinational data from the Ovarian Cancer Association Consortium’s (OCAC)^36^ online GWAS data set. Here, data for the *APOBEC3A/B*-deletion itself, was not available since all data were based on SNPs. Instead, we mined data for SNP rs12628403, previously shown to be in strong linkage disequilibrium with the deletion and therefore has been used previously as a surrogate marker for deletion status^27,35^. In the OCAC data set of > 18,000 cases and > 26,000 controls, allele-based data (not genotype data) was available. Although the OR estimates, here, were also < 1, the results were weaker and non-significant (OR = 0.97; CI = 0.92–1.02; Table 2, Fig. 2). We further assessed the available information on ovarian cancer subtypes and found a similar OR both when restricting the analyses to serous cancer and high-grade serous cancers (Table 2, Fig. 2). Data for the group of non-serous cancers in total was not available, but we mined information for the same three non-serous subtypes as assessed in our own data set. Again, the ORs were < 1, but in general, we observed weaker results than in our own data for all three subtypes (clear cell-, endometroid- and mucinous cancers). Notably, we observed a more significant association in the subgroup of clear cell cancers (OR = 0.85; CI = 0.69–1.00; Table 2, Fig. 2).

**Table 2:** SNP rs12628403* and risk estimates for ovarian cancer in OCAC.

OC subtype	Allele model
	OR (95% CI)
	d vs i
OCAC overall	0.97 (0.92–1.02) *p* = 0.26
Non-serous ovarian cancer	NA –
Clear cell ovarian cancer	0.85 (0.69–1.00) *p* = 0.04
Endometrioid ovarian cancer	0.97 (0.86–1.07) *p* = 0.54
Mucinous ovarian cancer	0.97 (0.86–1.08) *p* = 0.59
Serous ovarian cancer	0.99 (0.93–1.05) *p* = 0.71
High-grade serous ovarian cancer	0.99 (0.93–1.05) *p* = 0.66

*SNP rs12628403 used as a marker for the *APOBEC3A/B* deletion allele.

## Discussion

In the present study, we found the *APOBEC3A/B* deletion variant to be associated with a reduced risk of OC in the Norwegian population. Our findings were consistent across different models (dominant-, recessive- and allele-models), though our results from the recessive model should be interpreted with caution, given the low number of cases with homozygous del/del genotype.

In a previous study on the same population (Norwegians), we found the *APOBEC3A/B* deletion not to be associated with reduced risk of any of the four major cancer types, breast, prostate, lung- or colon cancer, in overall assessments^27^. However, we found a strongly reduced risk of lung cancer among young individuals and a highly significant trend-correlation for the ORs to change as a linear function of age. A similar, but non-significant linear trend was observed for prostate cancer. In the present analyses, we found the lowest OR among individuals at 60–69 years of age. As such, even though the OR for OC may be related to age, this does not follow the same linear trend as previously seen for lung- and prostate cancer.

Mining the large GWAS samples set from the OCAC consortium, we found the SNP rs12628403, which is strongly linked to the *APOBEC3A/B* deletion, to yield an OR below 1. Here, however, the OR was weaker than in our own data and the overall assessment did not reach statistical significance. The reasons for the discrepancies in OR between our in-house data and the OCAC-data remain unknown. However, it is worth noting that the MAF for the *APOBEC3A/B*-deletion varies across populations and it may be that the impact on risk is also variable with ethnicity. Notably, our in-house data set is exclusively based on Norwegian cases and controls^33^), while the OCAC sample set is merged from many different countries^36^. Further, our in-house data set was restricted to patients confirmed to have a *BRCA1/2* wild-type genotype, whereas the OCAC samples were unselected for BRCA-status.

The underlying risk factors for different subtypes of ovarian cancer are known to be different. This is most clearly exemplified by the fact that individuals with germline pathogenic mutations in *BRCA1* have a massively elevated risk of ovarian cancers of the high-grade serous subtype (HGSOC)^37^. Interestingly, the present data reveal a rather similar effect of the *APOBEC3A/B* deletion on the risk of the investigated subtypes. Both when restricting the analyses to serous cancers, and further to HGSOC, the risk estimates were similar to the overall estimate for all ovarian cancers in the study. For endometrioid ovarian cancers, we did find a risk reduction that was seemingly slightly more profound than in for other subtypes, but this risk reduction was not significantly different from the other estimates and the difference should therefore be interpreted with caution.

A main biological function of the APOBEC enzymes is to introduce mutations in viral nucleic acids entering the cell^14^. Recent advances in cancer genomics have revealed that the APOBEC enzymes also attack the cell’s own DNA and may cause bursts of mutations in the cancer genome^7,11^. The imprint of APOBEC activity in the cancer genome is also reflected by unique mutational signatures. As such, it is clear that the result of APOBEC hyperactivity is contributing to the molecular evolution of cancers towards more malignant states and also contributes to providing a plethora of mutations from which cancer cells may gain properties such as resistance to therapy. In light of these functions, one may assume that germline variants causing an elevated APOBEC activity should cause increased cancer risk. While this has been shown in some studies^23–25,30^, there are now emerging data, showing the opposite effect; that the *APOBEC3A/B* deletion is actually linked to reduced risk of e.g. lung cancer^27^. While the potential causes for this remain unknown it is tempting to speculate that elevated APOBEC activity in normal cells may reduce the impact of viral infection and therefore may reduce the risk of potential virally induced carcinogenesis. Notably, APOBEC activity has also been linked to antibody diversification and one may thus speculate that a slight increase in APOBEC activity may lead to a more diversified and/or adaptable immune system providing better tumour suppression^38–40^. More recently, it has been found that APOBEC3A is able to induce RNA editing in monocytes and macrophages^41^, and it has been reported that APOBEC3A promotes M1 macrophage polarization^42^, further indicating roles for APOBEC activity in relation to immune response activity.

In the present study, we validated genotyping by use of the SNP rs12628403 and, in some cases with ambiguous results from the main analysis, the SNP-genotyping was used to conclude for individual genotype. Further, our validation-effort, mining data from the OCAC consortium was based on the summary results (allele-data) for this SNP. Although this may, in theory, have introduced a bias in our data, we regard this potential bias to be negligible: In our present analyses, we found no discrepancy between the two methods. This is in line with our previous findings of a recombination rate of 0.5%, in a larger sample set of Norwegians^27^ and also in line with Middlebrooks and colleagues’ findings in Europeans in general^35^. As such, potential misinterpretations in the few cases where SNP-genotyping guided interpretations of ambiguous qPCR-HRM results, should not affect the present main results.

In conclusion, we found the APOBEC3A/B deletion genotype to be associated with a reduced risk of OC among Norwegian women.

## Supplementary Information

Supplementary Information.
