# Supplementary material for: Impact of the APOBEC3A/B deletion polymorphism on risk of ovarian cancer
Source: Sci Rep. 2021 Dec 6;11:23463. doi: 10.1038/s41598-021-02820-z (PMC8648731; doi:10.1038/s41598-021-02820-z)
Supplement: Supplementary file 1 — Supplementary Information. [file 41598_2021_2820_MOESM1_ESM.pdf]

# Supplementary Information

# Impact of the *APOBEC3A/B* deletion polymorphism on risk of ovarian cancer

Liv B. Gansmo[1,2], Nigar Sofiyeva[1,2], Merete Bjørnslett[3,4], Pål Romundstad[5], Kristian Hveem[6], Lars Vatten[5], Anne Dørum[7], Per E. Lønning[1,2] and Stian Knappskog[1,2,*]

[1]K.G.Jebsen Center for Genome-Directed Cancer Therapy, Department of Clinical Science, University of Bergen, Bergen, Norway.

[2]Department of Oncology, Haukeland University Hospital, Bergen, Norway.

[3]Department of Molecular Oncology, Oslo University Hospital Radium Hospitalet, Oslo, Norway.

[4]Institute for Cancer Research, University of Oslo, Oslo, Norway.

[5]Department of Public Health, Faculty of Medicine, Norwegian University of Science and Technology, Trondheim, Norway.

[6]K.G. Jebsen Center for Genetic Epidemiology, Department of Public Health, Faculty of Medicine, Norwegian University of Science and Technology, Trondheim, Norway.

[7]Department of Gynecologic Oncology, Oslo University Hospital, Norwegian Radium Hospital, Oslo, Norway.

* Corresponding author: Stian Knappskog, K.G.Jebsen Center for Genome-Directed Cancer Therapy, Department of Clinical Science, University of Bergen, 5021 Bergen, Norway. Tel: (+47) 55976447; Fax: (+47) 55972046; E-mail: stian.knappskog@uib.no, ORCID-ID: 0000-0002-4153-1655

**Supplementary Table S1**. Primer and probes used for *APOBEC3A/B* ins del genotyping

| Allele | Name | Primer/probe | Sequence 5' to 3' | Reference |
|---|---|---|---|---|
| Wild type | APO_ins2_F | Primer | TGTCCCTTTTCAGAGTTTGAGTA | 20 |
| | APO_ins2_R | Primer | TGGAGCCAATTAATCACTTCAT | 20 |
| | Apobec_ins2_acptr | Probe | LC-640-TGTCCCAGCAGTACTCAAACT-PH | 27 |
| | Apobec_ins2_donr | Probe | CATCCCTGGCGGTACACAA-FL | 27 |
| Deletion | APO_del_F | Primer | TAGGTGCCACCCCGAT | 20 |
| | APO_del_R | Primer | TTGAGCATAATCTTACTCTTGTAC | 20 |
| | Apobec_del_acptr | Probe | LC-640-TGTCCCAGCAGTGCTTAAATT-PH | 27 |
| | Apobec_del_donr | Probe | CATCCCTGGTGGTCCACAA-FL | 27 |

**Supplementary Table S2.** APOBEC3A/B genotype distribution and risk estimates for ovarian cancer in patients stratified by age groups.

| | | | | **Dominant model** | **Recessive model** | **Allele model** |
|---|---|---|---|---|---|---|
| **Age groups** | Genotype *APOBEC3A/B* n (%) | | | OR (95% CI) | OR (95% CI) | OR (95% CI) |
| | **ii** [1] | **id** [2] | **dd** [3] | **dd+id vs ii** | **dd vs id+ii** | **d vs i** |
| **< 50** | 186 (85.3) | 32 (14.7) | 0 (0.0) | 1.02 (0.64-1.59)<br>$p$ = 0.925 | NA<br>- | 0.98 (0.63-1.49)<br>$p$ = 0.920 |
| **50 – 59** | 321 (84.9) | 56 (14.8) | 1 (0.3) | 0.63 (0.43-0.93)<br>$p$ = 0.016 | 0.14 (0.00-1.08)<br>$p$ = 0.030 | 0.61 (0.43-0.88)<br>$p$ = 0.006 |
| **60 – 69** | 382 (87.2) | 54 (12.3) | 2 (0.5) | 0.51 (0.34-0.95)<br>$p = 4x10^{-4}$ | 0.31 (0.03-1.93)<br>$p$ = 0.146 | 0.52 (0.36-0.75)<br>$p = 3x10^{-4}$ |
| **70 – 79** | 259 (86.6) | 38 (12.7) | 2 (0.7) | 0.77 (0.49-1.19)<br>$p$ = 0.217 | 0.93 (0.08-8.18)<br>$p$ = 0.938 | 0.79 (0.52-1.19)<br>$p$ = 0.240 |
| **>80** | 55 (84.6) | 10 (15.4) | 0 (0.0) | 1.06 (0.33-3.59)<br>$p$ = 0.906 | NA<br>- | 1.06 (0.35-3.41)<br>$p$ = 0.910 |

[1] Genotype ins-ins
[2] Genotype ins-del
[3] Genotype del-del

Supplementary Fig. S1

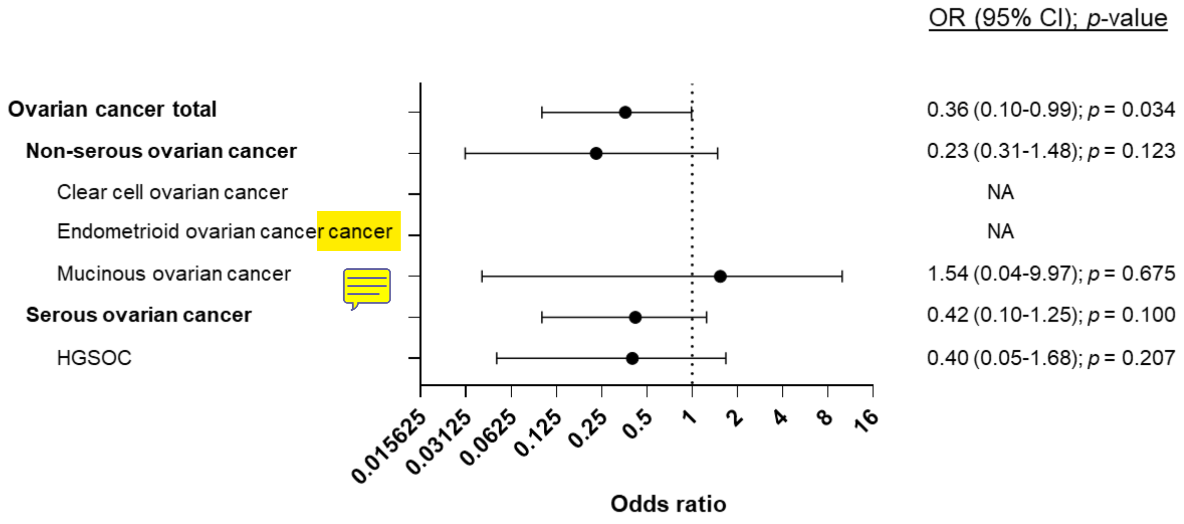


**Supplementary Fig. S1.** ***APOBEC3A/B*** **deletion and ovarian cancer risk; recessive model.** Forest plot illustrating odds ratios (ORs) with 95% confidence intervals (CI) for ovarian cancer and subtypes applying the recessive model.

Supplementary Fig. S2

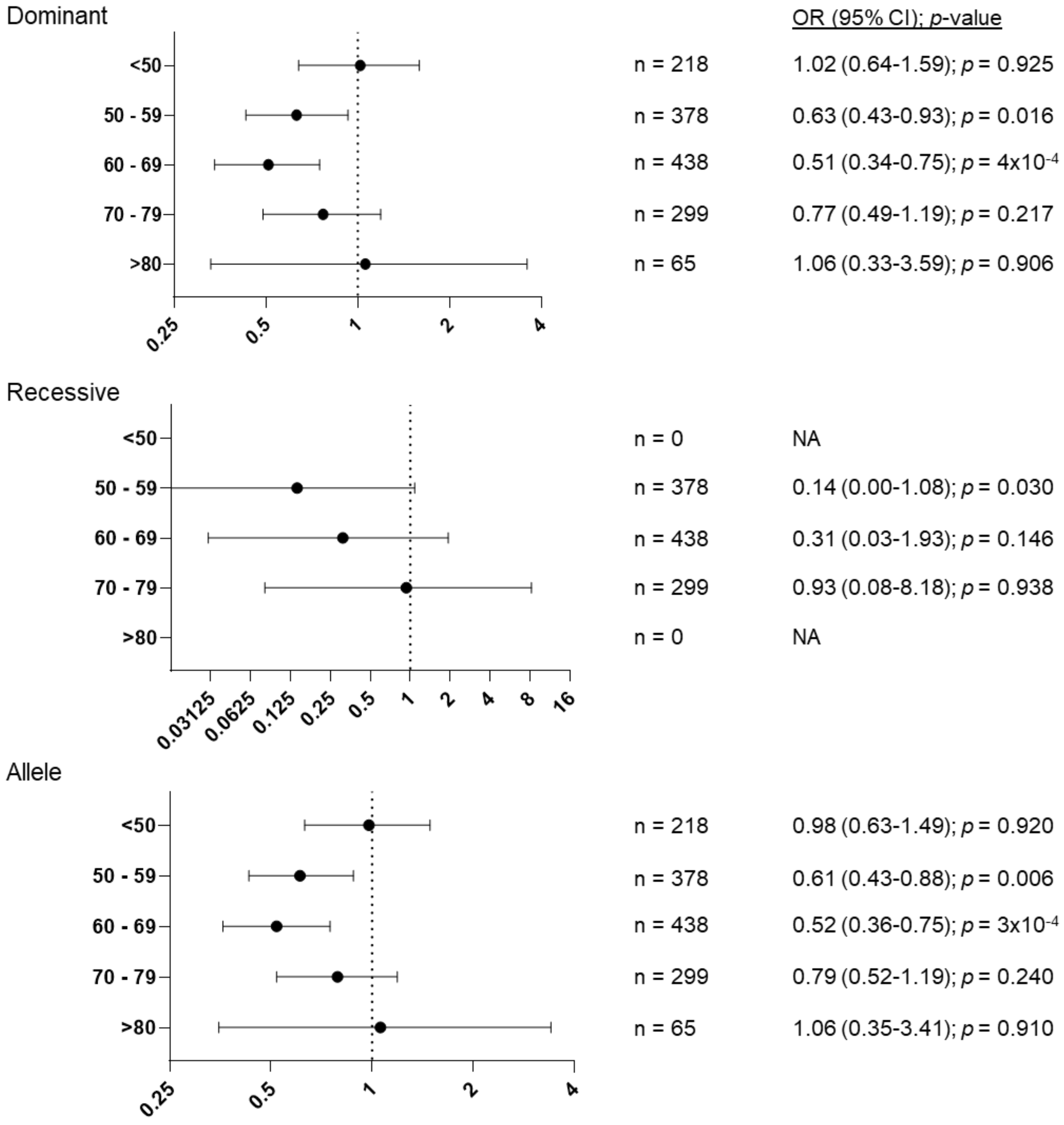


**Supplementary Fig. S2. *APOBEC3A/B* deletion variant and age-related cancer risk.** Forest plots depicting odds ratios (ORs) with 95% confidence intervals (CI) for ovarian cancer and subtypes related to the *APOBEC3A/B* deletion variant within age groups of 10 years intervals. Upper panel: dominant model; middle panel: recessive model and lower panel: allele model.
